# Effects of Sodium-Glucose Co-Transporter-2 Inhibitors on Markers of Vascular Damage

**DOI:** 10.3390/jpm13030536

**Published:** 2023-03-16

**Authors:** Christodoula Kourtidou, Vasileios Rafailidis, Garyfallia Varouktsi, Efthimios Kanakis, Vassilios Liakopoulos, Timoleon-Achilleas Vyzantiadis, Christos Savopoulos, Smaragdi Marinaki, Maria Stangou, Konstantinos Tziomalos

**Affiliations:** 1 First Propedeutic Department of Internal Medicine, Medical School, Aristotle University of Thessaloniki, AHEPA Hospital, 54636 Thessaloniki, Greece; 2 Department of Radiology, Medical School, Aristotle University of Thessaloniki, AHEPA Hospital, 54636 Thessaloniki, Greece; 3 Department of Nephrology, Medical School, Aristotle University of Thessaloniki, AHEPA Hospital, 54636 Thessaloniki, Greece; 4 First Department of Microbiology, Medical School, Aristotle University of Thessaloniki, 54124 Thessaloniki, Greece; 5 Department of Nephrology and Renal Transplantation, Medical School, National and Kapodistrian University of Athens, Laiko Hospital, 11527 Athens, Greece; 6 Department of Nephrology, Medical School, Aristotle University of Thessaloniki, Hippokration Hospital, 54642 Thessaloniki, Greece

**Keywords:** sodium–glucose co-transporter-2 inhibitors, diabetes mellitus, diabetic kidney disease, arterial stiffness, carotid atherosclerosis, peripheral arterial disease, ankle-brachial index, augmentation index, pulse wave velocity, carotid intima-media thickness

## Abstract

Background: Sodium glucose co-transporter 2 (SGLT2) inhibitors reduce cardiovascular morbidity and delay the progression of kidney disease in patients with type 2 diabetes mellitus (T2DM). However, the mechanisms underpinning these benefits are not entirely clear. More specifically, it is uncertain whether these agents exert cardiorenal protective effects through a direct action on the vascular wall. The aim of the present study was to evaluate the effects of SGLT2 inhibitors on markers of subclinical vascular damage. Methods: In total, 40 adult patients with T2DM and glomerular filtration rate (GFR) < 60 mL/min/1.73 m^2^ and age- and gender-matched patients with T2DM and GFR > 60 mL/min/1.73 m^2^ were consecutively enrolled. Indices of arterial stiffness (pulse wave velocity, augmentation index (AIx), AIx adjusted to a heart rate of 75 beats/min (Alx@75) and central systolic, diastolic, pulse and mean pressure), carotid atherosclerosis (stenosis, intima-media thickness (cIMT) and maximal plaque thickness) and peripheral arterial disease (ankle brachial index (ABI)) were determined. The chi-squared and Mann–Whitney U-test were used to detect differences in categorical and continuous variables between groups, respectively. Results: In total, 15 patients were treated with SGLT2 inhibitors and 25 patients were not receiving these agents. Serum low-density lipoprotein cholesterol levels were lower in the former whereas other cardiovascular risk factors, the prevalence of established cardiovascular disease, anthropometric and demographic characteristics, and vital signs did not differ between the 2 groups. The AIx was lower in patients treated with SGLT2 inhibitors (21.9 ± 11.3 vs. 29.7 ± 12% in patients not treated with SGLT2 inhibitors; *p* < 0.05). The AIx@75 was also lower in the former (21.3 ± 10.9 and 32.6 ± 11.3%, respectively, *p* < 0.005). Other markers of arterial stiffness were similar in the 2 groups. In addition, markers of carotid atherosclerosis and the ABI did not differ between patients treated and not treated with SGLT2 inhibitors. Conclusions: Treatment with SGLT2 inhibitors appears to reduce arterial stiffness. Accordingly, these agents might improve cardiovascular outcomes not only in patients with T2DM and established cardiorenal disease but also in lower-risk patients.

## 1. Introduction

Sodium glucose co-transporter 2 (SGLT2) inhibitors are the antidiabetic agents of choice, along with glucagon-like peptide 1 receptor agonists, in patients with type 2 diabetes mellitus (T2DM) who also have established atherosclerotic cardiovascular disease (CVD), are at high risk for CVD, have established heart failure (HF) and/or have chronic kidney disease (CKD) [1,2]. Notably, SGLT2 inhibitors should be considered in these patients independently of baseline HbA_1c_, individualized HbA_1c_ target and the use of metformin [1,2]. These recommendations are based on the findings of several randomized clinical trials that showed a beneficial effect of SGLT2 inhibitors on cardiovascular and renal outcomes [3,4,5]. Indeed, SGLT2 inhibitors reduce the risk for hospitalization for heart failure and delay the progression of diabetic kidney disease [3].

The mechanisms underpinning the salutary cardiorenal actions of SGLT2 inhibitors are not fully clarified [6,7]. In addition to glucose- and blood pressure-lowering and natriuretic effects, a direct action on the vascular wall has been suggested to play a role in the reduction in cardiovascular and renal morbidity conferred by these agents [8,9,10,11]. However, data on the effects of SGLT2 inhibitors on subclinical vascular disease, including arterial stiffness, carotid atherosclerosis and peripheral arterial disease (PAD) are conflicting and limited [12,13,14,15,16]. Whether SGLT2 inhibitors exert beneficial effects in patients without established CVD but with subclinical vascular disease is particularly pertinent, given the increased cardiovascular risk of this patient population [17,18,19]. Moreover, even though SGLT2 inhibitors reduce the risk of atherosclerotic cardiovascular events in patients with established CVD, this benefit is less clear in patients without CVD [3].

The aim of the present study was to evaluate the effects of SGLT2 inhibitors on arterial stiffness, carotid atherosclerosis and PAD.

## 2. Materials and Methods

Methods have been reported in detail previously [20]. In brief, this is a prespecified analysis of a cross-sectional study in which we enrolled all adult patients with diabetic kidney disease (DKD) [21] who visited the Internal Medicine Outpatients Clinic of the First Propedeutic Department of Internal Medicine between August 2021 and April 2022. Age- and gender-matched patients with T2DM but without DKD who visited the same outpatient clinic during this period were enrolled as controls. Demographic data, presence of cardiovascular risk factors or established cardiovascular disease, anthropometric parameters and vital signs were recorded. Lipid profile, HbA_1c_ and creatinine were measured in fasting blood samples and the urinary albumin/creatinine ratio was determined in a morning spot urine sample. SGLT2 inhibitors were prescribed according to current guidelines, i.e., were administered to all patients with HF and/or CKD in the absence of contraindications and were also an option in patients who could not achieve HbA_1c_ targets despite treatment with metformin [1,2].

The following indices of arterial stiffness were measured using the Sphygmocor device (Atcor Medical, Sydney, Australia): pulse wave velocity (PWV), augmentation index (AIx), AIx adjusted to a heart rate of 75 beats/min (Alx@75) and central systolic, diastolic, pulse and mean pressure [22]. Stenosis, intima-media thickness (cIMT) and maximal plaque thickness were measured in both carotid arteries using a Logiq S8 ultrasound device (GE Healthcare, Milwaukee, Wisconsin) according to current guidelines [23,24,25]. The ankle brachial index (ABI) was measured in both legs to determine the presence of PAD [26].

IBM SPSS Statistics for Windows (version 27: IBM Corp: Armonk, NY, USA) was used for statistical analysis. The chi-square test was used to detect differences in categorical variables between groups. The Mann–Whitney U-test was used to detect differences in continuous variables between groups.

The study was conducted according to the Declaration of Helsinki and was approved by the Ethics Committee of the Medical School of the Aristotle University of Thessaloniki. All patients provided written informed consent.

## 3. Results

Forty patients with T2DM were enrolled in the study (Table 1). Fifteen patients were treated with SGLT2 inhibitors (73.3% males, age 68.9 ± 7.3 years) and 25 patients were not treated with SGLT2 inhibitors (68% males, age 73.2 ± 9.6 years). The mean duration of treatment with SGLT2 inhibitors was 3.6 ± 1.2 years. Among the 15 patients who were treated with SGLT2 inhibitors, 13patients were receiving empagliflozin and 2 patients were receiving dapagliflozin. Serum low-density lipoprotein cholesterol (LDL-C) levels were lower in patients treated with SGLT2 inhibitors (43.1 ± 19.1 vs. 67.8 ± 46.9 mg/dL in patients not treated with SGLT2 inhibitors; *p* < 0.05). Other cardiovascular risk factors, the prevalence of established CVD, anthropometric and demographic characteristics, and vital signs did not differ between the twogroups.

Markers of subclinical vascular disease according to treatment with SGLT2 inhibitors are shown in Table 2. The AIx was lower in patients treated with these agents (21.9 ± 11.3 vs. 29.7 ± 12% in patients not treated with SGLT2 inhibitors; *p* < 0.05). The AIx@75 was also lower in the former (21.3 ± 10.9 and 32.6 ± 11.3%, respectively, *p*< 0.005). Other markers of arterial stiffness (PWV, cSBP, cDBP, cPP and cMP) were similar in the 2 groups. cIMT of the left carotid artery showed a trend for being lower in patients treated with SGLT2 inhibitors (0.07 ± 0.02 vs. 0.09 ± 0.02 mm in patients not treated with SGLT2 inhibitors; *p* = 0.065). Other markers of carotid atherosclerosis (stenosis and maximal plaque thickness) did not differ between the 2 groups. The ABI was also similar in both legs in patients treated and not treated with SGLT2 inhibitors.

One of the patients who received SGLT2 inhibitors developed a urinary tract infection which was managed with antibiotic therapy and did not require discontinuation of the SGLT2 inhibitor. No other adverse events were observed in the twogroups.

## 4. Discussion

In the present study, AΙx and AΙx@75, which are surrogate markers of arterial stiffness [27], were lower in patients receiving SGLT2 inhibitors than in those not treated with these agents. Notably, age and the prevalence of hypertension, which are the major risk factors promoting arterial stiffening, did not differ between these 2 groups [22]. In accordance with our findings, a reduction in AIx@75 was observed after 12 weeks of treatment with dapaglifozin in a recent study in 44 patients with T2DM [12]. Similarly, AIx and AIx@75 improved after 8 weeks of empaglifozin administration in 40 normotensive patients with type 1 diabetes [13]. Given the association between arterial stiffness and cardiovascular morbidity and mortality [17,28,29], the improvement in arterial stiffness with SGLT2 inhibitors might contribute to the reduced risk for cardiovascular events during treatment with these agents. In contrast to these findings, AIx and AIx@75 did not change after 2 days of dapaglifozin treatment in another study in 16 patients with T2DM [14]. However, the small size and the short duration of treatment might explain these negative results [14].

We observed a trend for lower cIMT in the left carotid artery in patients treated with SGLT2 inhibitors, where other markers of carotid atherosclerosis were not affected by this treatment. There are limited and conflicting data on the effects of SGLT2 inhibitors on carotid atherosclerosis. In twostudies in Japanese patients, tofogliflozin reduced cIMT to a similar degree with other antidiabetic agents [15], whereas SGLT2 inhibitors had no effect on cIMT in patients with T2DM and heart failure [16]. Notably, treatment with SGLT2 inhibitors was shown to stabilize carotid plaque in patients with T2DM [30]. Given the paucity of the data, further research is needed to clarify the role of SGLT2 inhibitors in delaying the progression of carotid atherosclerosis.

In our study, the ABI in both legs did not differ between patients who were receiving SGLT2 inhibitors and those who were not. To the best of our knowledge, this is the first study that evaluated the effects of SGLT2 inhibitors on ABI in patients with T2DM. These findings are potentially important, given the increased risk for lower limb amputation in patients treated with canagliflozin [31,32]. On the other hand, other SGLT2 inhibitors do not appear to increase the risk of adverse lower limb events in patients with PAD [31,32,33,34]. Nevertheless, more studies are needed to evaluate the effects of SGLT2 inhibitors on PAD, given its high prevalence in patients with T2DM and its association with increased risk for cardiovascular events [18].

The major limitation of our study is that it is a cross-sectional analysis of the association between treatment with SGLT2 inhibitors and the presence of subclinical vascular disease in a small number of patients. Therefore, our study cannot provide definitive evidence of a protective effect of these agents against vascular damage. Indeed, it is possible that patients who were at higher risk for vascular disease were preferentially treated with SGLT2 inhibitors. However, there were no differences in cardiovascular risk factors between patients treated and not treated with these agents (except for LDL-C levels, which were lower in the former) suggesting that SGLT2 inhibitors might indeed protect agains vascular damage. In addition, there are very few adequately designed, large, randomized, placebo-controlled studies that evaluated the effects of these agents on arterial stiffness, carotid atherosclerosis and subclinical PAD and therefore our study might be a potentially useful addition to this research field. On the other hand, our findings are too preliminary to be translated to changes in the current management of patients with T2DM and must be validated in more rigorously designed and larger studies.

## 5. Conclusions

Treatment with SGLT2 inhibitors appears to reduce arterial stiffness and also tends to reduce the atherosclerotic burden in the carotid arteries. Accordingly, these agents might improve cardiovascular outcomes not only in patients with T2DM and established CVD but also in lower-risk patients. More specifically, patients with T2DM and increased arterial stiffness and/or carotid atherosclerosis might represent potential candidates for treatment with SGLT2 inhibitors. However, more data are needed to define the role of SGLT2 inhibitors in the management of patients with T2DM without clinically evident atherosclerosis.

## Figures and Tables

**Table 1:** Characteristics of patients treated and not treated with sodium-glucose co-transporter 2 (SGLT2) inhibitors (cases and controls, respectively).

	Cases	Controls	*p*
	(*n* = 15)	(*n* = 25)	
Age (years)	68.9 ± 7.3	73.2 ± 9.6	NS
Males (%)	73.3	68.0	NS
Systolic blood pressure (mmHg)	138.1 ± 14.2	142.8 ± 14.6	NS
Diastolic blood pressure (mmHg)	81.9 ± 10.5	81.7 ± 14.7	NS
Heart rate	70.6 ± 10.6	74.7 ± 9.9	NS
Hypertension (%)	86.7	88.0	NS
Hypertension duration (years)	15.9 ± 8.3	17.5 ± 6.5	NS
Type 2 diabetes mellitus duration (years)	12.6 ± 9.1	13.3 ± 7.1	NS
Smoking (current/past, %)	20.0/40.0	12.0/40.0	NS
Package-years	57.5 ± 37.1	37.7 ± 22.7	NS
Alcohol intake (units/week)	0.8 ± 1.4	0.7 ± 1.6	NS
Atrial fibrillation (%)	13.3	32.0	NS
Family history of cardiovascular disease (%)	6.7	32.0	NS
Coronary heart disease (%)	40.0	40.0	NS
Prior ischemic stroke (%)	0.0	4.0	NS
Heart failure (%)	13.3	20.0	NS
Weight (kg)	80.7 ± 10.4	84.6 ± 19.2	NS
Body mass index (kg/m^2^)	28.1 ± 3.4	29.4 ± 5.7	NS
Waist circumference (cm)	102.8 ± 7.9	107.4 ± 13.2	NS
Total cholesterol (mg/dL)	112.7 ± 21.9	134.3 ± 55.5	NS
Low-density lipoprotein cholesterol (mg/dL)	43.1 ± 19.1	67.8 ± 46.9	<0.05
High-density lipoprotein cholesterol (mg/dL)	42.1 ± 11.4	41.9 ± 19.1	NS
Triglycerides (mg/dL)	136.6 ± 48.8	147.7 ± 77.4	NS
HbA_1c_ (%)	7.1 ± 0.7	7.4 ± 1.5	NS
Chronic kidney disease (%)	33.3	52.0	NS
Estimated glomerular filtration rate (mL/min/1.73m^2^)	72.3 ± 25.4	59.7 ± 28.5	NS
Urinary albumin/creatinine ratio (mg/g)	94.3 ± 127.4	181.9 ± 415.3	NS
Lipid-lowering treatment (%)	100.0	100.0	NS
Antihypertensive treatment (%)	86.7	88.0	NS
Antidiabetic treatment (%)			
Metformin	93.3	92.0	NS
Dipeptidyl peptidase-4 inhibitors	33.3	48.0	NS
Insulin	20.0	24.0	NS
Other	13.3	12.0	NS

**Table 2:** Markers of subclinical vascular disease in patients treated and not treated with sodium-glucose co-transporter 2 (SGLT2) inhibitors (cases and controls, respectively).

	Cases	Controls	*p*
	(*n* = 15)	(*n* = 25)	
Ankle-brachial index (left)	1.17 ± 0.21	1.03 ± 0.26	NS
Ankle-brachial index (right)	1.09 ± 0.21	1.06 ± 0.26	NS
Augmentation index (%)	21.9 ± 11.3	29.7 ± 12.0	<0.05
Augmentation index @75 (%)	21.3 ± 10.9	32.6 ± 11.3	<0.005
Pulse wave velocity (m/sec)	7.2 ± 4.7	8.6 ± 5.3	NS
Central systolic blood pressure (mmHg)	125.9 ± 11.8	128.9 ± 10.3	NS
Central diastolic blood pressure (mmHg)	79.4 ± 11.6	78.8 ± 13.1	NS
Central mean blood pressure (mmHg)	99.9 ± 11.4	100.9 ± 11.9	NS
Central pulse pressure (mmHg)	46.5 ± 15.3	48.6 ± 12.4	NS
Carotid stenosis (left)(%)	31.7 ± 18.2	29.7 ± 14.9	NS
Carotid intima-media thickness (left)(cm)	0.07 ± 0.02	0.09 ± 0.02	NS
Maximal plaque thickness (left)(cm)	0.20 ± 0.14	0.22 ± 0.10	NS
Carotid stenosis (right)(%)	21.5 ± 18.1	31.5 ± 17.9	NS
Carotid intima-media thickness (right)(cm)	0.08 ± 0.02	0.09 ± 0.03	NS
Maximal plaque thickness (right)(cm)	0.23 ± 0.29	0.23 ± 0.11	NS
Carotid intima-media thickness ≥ 0.1 cm on either side (%)	33.3	60.0	NS

## Data Availability

Data are available upon request.
